# Mitotic Spindle Positioning (MISP) Facilitates Colorectal Cancer Progression by Forming a Complex with Opa Interacting Protein 5 (OIP5) and Activating the JAK2-STAT3 Signaling Pathway

**DOI:** 10.3390/ijms25053061

**Published:** 2024-03-06

**Authors:** Koki Hiura, Masaki Watanabe, Naoki Hirose, Kenta Nakano, Tadashi Okamura, Hayato Sasaki, Nobuya Sasaki

**Affiliations:** 1 Laboratory of Laboratory Animal Science and Medicine, School of Veterinary Medicine, Kitasato University, Towada 034-8628, Japan; 2 The Institute of Experimental Animal Sciences, Faculty of Medicine, Osaka University, Osaka 565-0871, Japan; 3 Department of Laboratory Animal Medicine, Research Institute, National Center for Global Health and Medicine, Tokyo 162-8655, Japan

**Keywords:** azoxymethane, dextran sodium sulfate, colorectal cancer, MISP, OIP5, JAK2-STAT3 pathway

## Abstract

Patients with inflammatory bowel disease (IBD) who experience long-term chronic inflammation of the colon are at an increased risk of developing colorectal cancer (CRC). Mitotic spindle positioning (MISP), an actin-binding protein, plays a role in mitosis and spindle positioning. MISP is found on the apical membrane of the intestinal mucosa and helps stabilize and elongate microvilli, offering protection against colitis. This study explored the role of MISP in colorectal tumorigenesis using a database, human CRC cells, and a mouse model for colitis-induced colorectal tumors triggered by azoxymethane (AOM)/dextran sodium sulfate (DSS) treatment. We found that MISP was highly expressed in colon cancer patient tissues and that reduced MISP expression inhibited cell proliferation. Notably, MISP-deficient mice showed reduced colon tumor formation in the AOM/DSS-induced colitis model. Furthermore, MISP was found to form a complex with Opa interacting protein 5 (OIP5) in the cytoplasm, influencing the expression of OIP5 in a unidirectional manner. We also observed that MISP increased the levels of phosphorylated STAT3 in the JAK2-STAT3 signaling pathway, which is linked to tumorigenesis. These findings indicate that MISP could be a risk factor for CRC, and targeting MISP might provide insights into the mechanisms of colitis-induced colorectal tumorigenesis.

## 1. Introduction

Colorectal cancer (CRC) is the third leading cause of tumor-related deaths worldwide [1]. The majority of human CRC cases are not caused by hereditary genetic changes but rather by environmental risk factors such as chronic inflammation, food-associated mutagens, and specific intestinal microbiota [2]. Among these environmental risk factors, chronic inflammation is the most significant risk factor for CRC carcinogenesis. Furthermore, patients diagnosed with inflammatory bowel diseases (IBD) such as ulcerative colitis (UC) and Crohn’s disease (CD) have a significantly higher risk of developing colitis-associated CRC (CAC) and a higher mortality rate compared to other CRC patients [3,4,5]. Additionally, tumor-related inflammation was observed in tissue samples from a large population of patients who showed no signs of IBD before the initial stages of CRC, suggesting that inflammation plays a crucial role in the development of CRC [6]. The JAK2-STAT3 signaling pathway is a critical inflammatory pathway, and IBD is associated with abnormal STAT3 regulation. Constitutively activated STAT3 translocates to the nucleus, where it enhances transcription factors and promotes cell proliferation, resistance to apoptosis, survival, angiogenesis, and tumor-promoting inflammation [7,8]. Experimental mouse models of CRC have been created by pioneers in the field, providing critical insights into the mechanisms of CRC development, drug discovery, and the identification of new therapeutic targets [9,10]. The azoxymethane (AOM)/dextran sodium sulfate (DSS)-induced CAC mouse model is one of the most widely used chemical CRC models due to its high reproducibility and efficacy. Research based on this model has demonstrated the importance of the inflammatory process in CRC development and has shed light on some of the mechanisms of inflammation-related colorectal carcinogenesis in the gut, with a focus on the functions of inflammatory and anti-inflammatory cytokines [9,11]. In the field of UC drug development, the nature of the disease as an IBD has traditionally focused on targets related to the immune system. However, recognizing that UC is a multifactorial disease affected by various cellular processes, it is expected that research exploring molecular targets beyond the immune system will increase in the future [12].

Mitotic spindle positioning (MISP) is a protein that contains multiple actin-binding sites [13]. MISP has been reported to be involved in both mitotic progression and spindle positioning [14,15]. It is highly localized to the microvillar rootlets in the apical membrane of the intestinal mucosa and has also been shown to stabilize and elongate the rootlet ends of core actin bundles in microvilli [16]. ARCHS4 (https://maayanlab.cloud/archs4/, accessed on 15 January 2021) suggests that the predicted biological processes of MISP include intestinal absorption and sulfate transmembrane transport [17]. In our previous study, generalized *Misp* knockout (KO) mice were generated using CRISPR/Cas9-mediated genome editing. *Misp* KO mice were healthy and showed no histological abnormalities in any of their organs, including the intestine. However, *Misp* KO mice have been found to exhibit significant exacerbation of colitis symptoms, such as weight loss and loss of crypt tissue, in DSS-induced colitis, suggesting that MISP plays a protective role against colitis [18]. MISP is a substrate of Polo-like kinase 1 (PLK1) and is phosphorylated by PLK1 to form a complex with the +TIP-binding protein p150^glued^, which is a subunit of the dynein–dynactin complex. This complex is involved in spindle positioning and mitotic regulation by influencing the distribution of p150^glued^ in the cell cortex [15]. The regulation of p150^glued^ localization in the cell cortex suggests that MISP interacts with the multi-domain protein IQGAP1, contributing to IQGAP1-mediated Cdc42 activation [19]. While the mechanisms of MISP in mitosis are being elucidated, the signaling pathways associated with MISP remain largely unresolved, and its role in CRC has yet to be elucidated.

In this study, we hypothesized that MISP promotes the development of CRC and investigated the relationship between MISP and intestinal tumorigenesis by evaluating tumor development induced by colitis in *Misp* KO mice using the AOM/DSS model. Notably, we observed a reduction in the occurrence of colitis-induced colorectal tumors in *Misp* KO mice, which exhibited intensified inflammation after DSS treatment. Furthermore, we analyzed factors associated with MISP and tumorigenesis in human CRC cell lines. Therefore, a thorough analysis of MISP function in colitis-induced colorectal tumorigenesis at the cellular level indicates that MISP is a risk factor for CRC.

## 2. Results

### 2.1. MISP Expression Is Elevated in Patients with CRC

**Figure 1:**
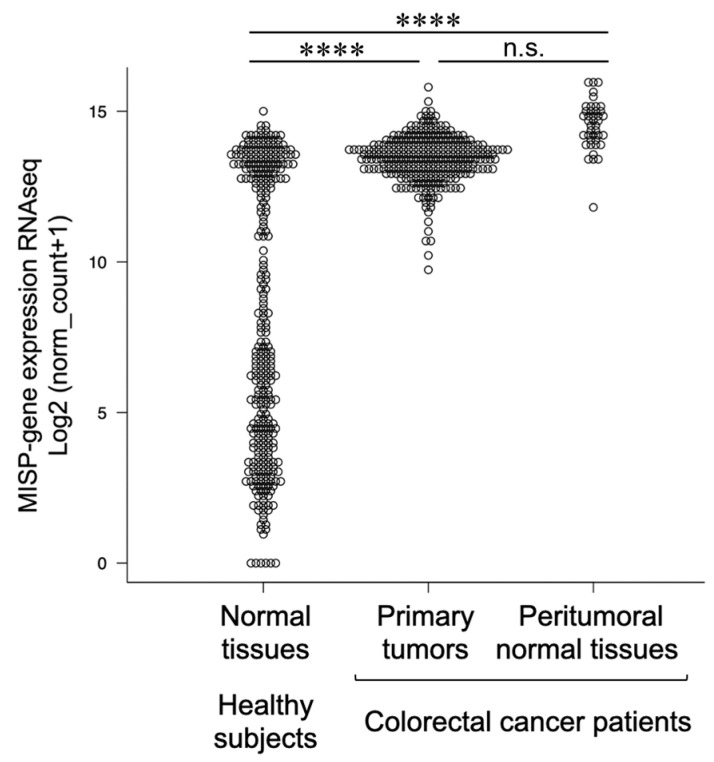
Comparison of the expression of the mitotic spindle positioning (MISP) gene in colorectal tissues. The Cancer Genome Atlas (TCGA) is a cancer genome database. The analysis includes normal tissue samples from healthy subjects (*n* = 308) and colorectal cancer (CRC) patients (primary tumors, *n* = 288; peritumoral normal tissues, *n* = 41). **** *p* < 0.0001; n.s., no significant difference.

The University of California Santa Cruz (UCSC) Xena Browser, which can be accessed at https://xenabrowser.net (accessed on 8 February 2021), facilitates the comparative analysis of RNA sequencing (RNA-seq) data between healthy individuals and cancer patients. This comparison utilizes the TCGA TARGET GTEx data set, which is available at https://ocg.cancer.gov/programs/target (accessed on 8 February 2021), from the Cancer Genome Atlas (TCGA), a comprehensive cancer genome repository [20,21,22]. Our investigation into the role of MISP in tumorigenesis involved examining alterations in its expression within normal and cancerous cells. We found a significant upregulation of MISP expression in the colonic tissues of CRC patients (in both primary tumors and adjacent normal tissues) compared with healthy controls. Moreover, a bimodal distribution pattern of MISP expression was observed in the colonic tissues of healthy individuals, with distinct high and low expression groups. Notably, there were no marked differences in the levels of MISP expression between primary tumors and the surrounding normal tissues in CRC patients. The subset of healthy individuals with higher MISP expression exhibited levels similar to those found in CRC patients (Figure 1).

### 2.2. MISP Enhances Cell Proliferation in Human CRC Cell Lines

**Figure 2:**
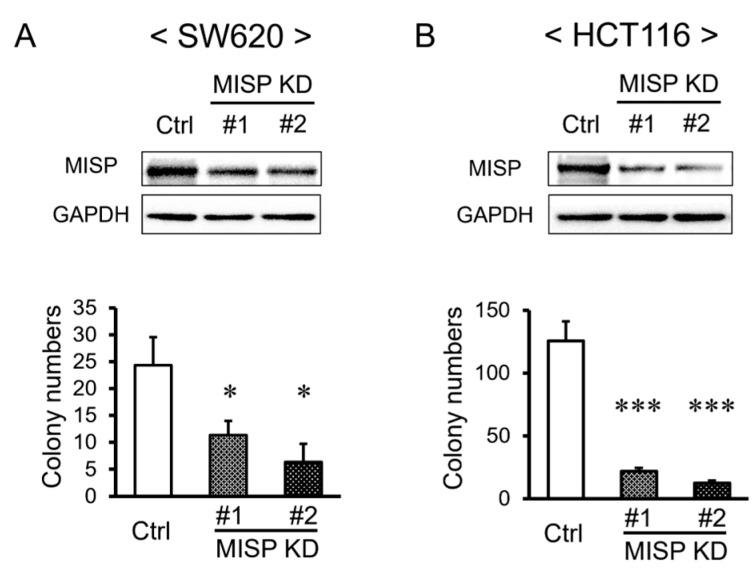
Effect of the decreased MISP expression on the proliferation of CRC cells. (**A**) Evaluation of the effect of MISP knockdown (KD) on the proliferation of SW620 cells. (**B**) Assessment of the effect of MISP knockdown on the proliferation of HCT116 cells. The experiments were conducted with three replicates (*n* = 3). * *p* < 0.05; *** *p* < 0.001.

The influence of MISP on cellular proliferation was assessed using a colony formation assay. The suppression of MISP expression (MISP knockdown, KD) led to a significant reduction in both the colony-forming capacity and proliferation rates in human CRC cell lines, SW620 and HCT116 (Figure 2).

### 2.3. Impact of MISP on Inflammation in a Mouse Model of Colitis-Induced Colon Tumors

**Figure 3:**
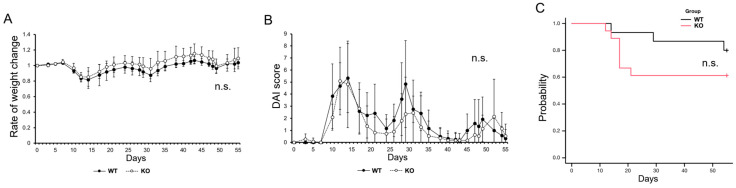
Evaluation of the impact of MISP on chronic inflammation of the colon in the azoxymethane (AOM)/dextran sodium sulfate (DSS) model. (**A**) Analysis of weight fluctuations. (**B**) Assessment of Disease Activity Index (DAI) scores. (**C**) Examination of the survival rate. In panels (**A**,**B**), the study included two groups: wild-type (WT, *n* = 11) and *Misp* knockout (KO, *n* = 12). In panel (**C**), the study involved two groups: WT (*n* = 15) and *Misp* KO (*n* = 18). No significant difference was observed (n.s.).

We evaluated weight changes, Disease Activity Index (DAI) scores, and survival rates in the AOM/DSS mouse model to examine the role of MISP in chronic inflammation. The DAI score was determined by combining three individual scores: the rate of weight loss, stool characteristics, and the severity of hematochezia, to assess the severity of colitis [18,23,24]. Our observations revealed no significant differences in the rates of weight change, DAI scores, or survival rates between *Misp* KO mice and their wild-type (WT) counterparts (Figure 3).

### 2.4. MISP Facilitates the Growth of Colitis-Induced Colorectal Tumors

**Figure 4:**
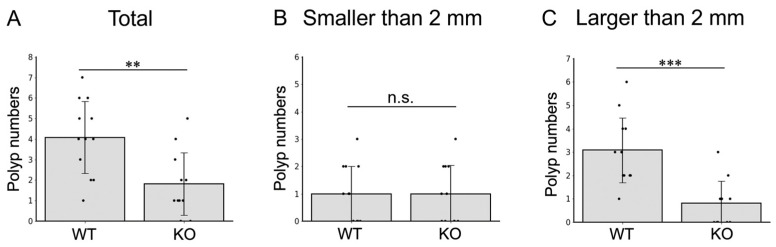
Investigation of the effect of MISP on the initiation of colitis-induced colorectal tumorigenesis in the AOM/DSS model. (**A**) Total number of tumors. (**B**) Number of tumors smaller than 2 mm. (**C**) Number of tumors larger than 2 mm. The study included two groups: WT (*n* = 11) and KO (*n* = 12). ** *p* < 0.01; *** *p* < 0.001; n.s., no significant difference. The number of polyps in each mouse is indicated by the dots.

In the AOM/DSS model for colitis-induced colorectal tumors, we measured tumor growth by counting the total number of tumors and assessing their sizes. The comparative analysis of colon tumors in WT and *Misp* KO mice revealed a significant reduction in both the total number of tumors and the number of tumors exceeding 2 mm in diameter in the *Misp* KO group. However, there was no significant difference in the incidence of tumors smaller than 2 mm (Figure 4).

### 2.5. Investigating Genes Associated with MISP in CRC

**Figure 5:**
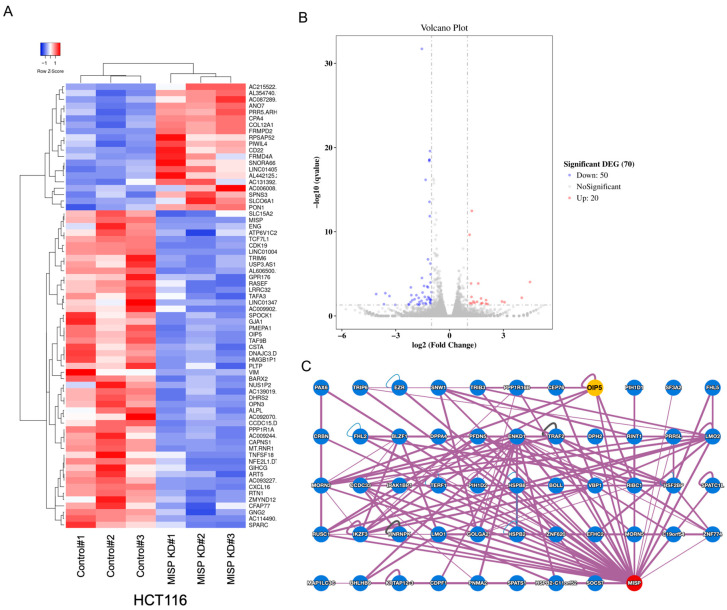
Exploration of MISP-related factors involved in colorectal tumorigenesis through RNA sequencing (RNA-seq) and database analysis. (**A**,**B**) Analysis of genes exhibiting significant expression changes in MISP-KD HCT116 cells using RNA-seq, presented as (**A**) a heat map and (**B**) a volcano plot. The analysis was conducted with three replicates (*n* = 3). (**C**) Exploration of proteins that potentially interact with MISP based on data from the human binary protein interactome database (HuRI).

To identify genes associated with MISP, we examined changes in gene expression resulting from reduced MISP levels using RNA-seq analysis. In the HCT116 human colorectal cancer cell line, we detected a substantial alteration in the expression levels of a total of 70 genes upon MISP knockdown compared to the control group. We set a stringent significance threshold of 0.1% for these changes (Figure 5A,B). Pathway analysis was performed using Enrichr (https://maayanlab.cloud/Enrichr/, accessed on 1 July 2021) and KEGG Mapper (https://www.genome.jp/kegg/mapper/, accessed on 15 July 2021) [25,26] on genes that exhibited significant expression changes due to MISP KD. No significant pathways thought to be involved in tumor proliferation were detected. The Human Binary Protein Interactome (HuRI), which can be accessed at http://www.interactome-atlas.org (accessed on 22 February 2022), is a compendium of native protein–protein interactions (PPIs) identified through the yeast two-hybrid (Y2H) screening technique [27]. To narrow down the 70 genes that showed significant expression changes upon MISP knockdown to those more suggestive of involvement with MISP, we used HuRI to identify proteins predicted to interact with MISP, including Opa Interacting Protein 5 (OIP5). Notably, OIP5 exhibited a significant change in expression in the MISP KD samples, as revealed in the RNA-seq data (Figure 5C).

### 2.6. Elevated Expression of OIP5 in Colorectal Cancer Patients and Its Correlation with MISP Expression in the Colon

**Figure 6:**
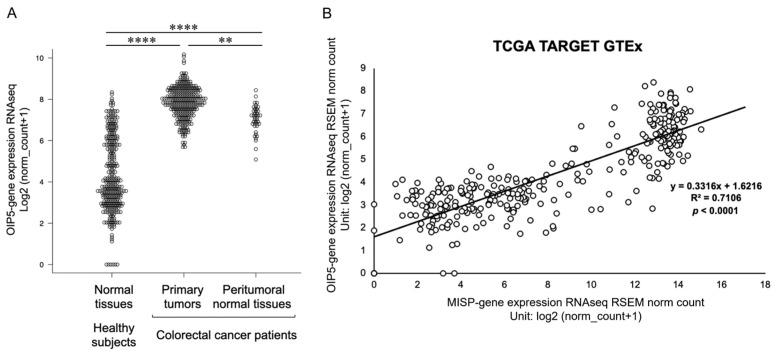
Comparison of Opa interacting protein 5 (OIP5) expression levels in colorectal tissues and correlation analysis of OIP5 and MISP expression. (**A**) Comparison of the expression levels of OIP5 in colorectal tissues between healthy individuals and CRC patients, analyzing primary tumors and adjacent normal tissues using data from TCGA. The analysis included normal tissues from healthy subjects (*n* = 308) and CRC patients (primary tumors, *n* = 288; peritumoral normal tissues, *n* = 41). ** *p* < 0.01; **** *p* < 0.0001. (**B**) Correlation analysis was conducted to assess the relationship between MISP and OIP5 expression in colon tissues from healthy subjects. The dataset included normal tissues from healthy individuals (*n* = 308).

Our research focused on the role of OIP5 in tumorigenesis by analyzing variations in OIP5 expression between normal and cancerous cells. We observed a significant increase in OIP5 expression levels in the colonic tissues of patients with CRC, both in primary tumors and adjacent normal tissues, compared with healthy individuals. The expression profile of OIP5 in the colonic tissues of healthy subjects varied widely, ranging from low to high levels. In CRC patients, OIP5 expression was notably elevated in primary tumors compared to the surrounding normal tissues (Figure 6A). Additionally, we assessed the correlation between OIP5 and MISP expression in the colon of healthy subjects using the TCGA TARGET GTEx dataset. The analysis revealed a strong correlation between the expression levels of OIP5 and MISP (Figure 6B).

### 2.7. MISP Influences OIP5 Expression through Complex Formation, While OIP5 Does Not Alter MISP Expression

**Figure 7:**
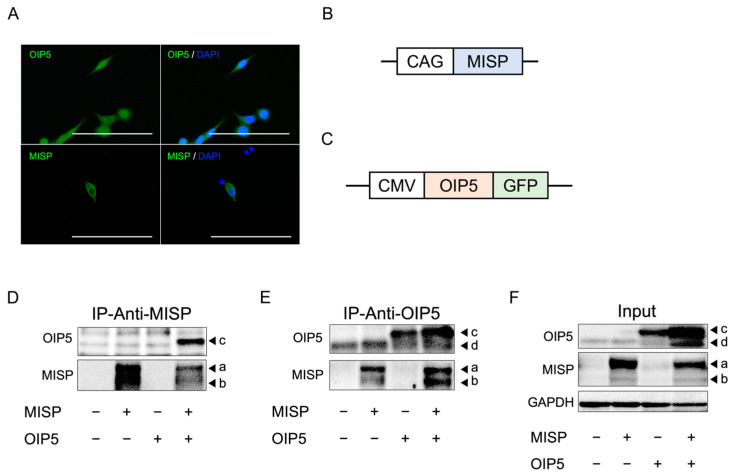
Verification of the interaction between MISP and OIP5. (**A**) Immunofluorescence staining was conducted to determine the localization of MISP and OIP5 in HCT116 cells. Scale bars, 50 µm. (**B**,**C**) Schematic diagrams illustrating the construction of expression plasmids for (**B**) MISP and (**C**) OIP5. (**D**–**F**) Analysis of the formation of MISP and OIP5 complexes using immunoprecipitation (IP) with (**D**) MISP and (**E**) OIP5 antibodies. (**F**) The input samples. Arrowheads a and b indicate MISP, while arrowheads c and d indicate OIP5, with c being exogenous and d being endogenous.

Immunofluorescence staining conducted on HCT116 cells to determine the subcellular localization of MISP and OIP5 revealed that MISP was localized in the cytoplasm, whereas OIP5 was present in both the nucleus and cytoplasm (Figure 7A). To examine whether MISP and OIP5 interact to form a complex, we overexpressed each protein in 293FT human fetal kidney cells using expression plasmids for MISP or OIP5 and performed immunoprecipitation and Western blot analyses. The MISP antibody identified overexpressed MISP in the input, while the endogenous MISP in 293FT cells was undetectable. The OIP5 antibody recognized both the GFP-tagged OIP5 and the untagged endogenous OIP5 in 293FT cells overexpressing OIP5. Immunoprecipitation with the MISP antibody resulted in the detection of OIP5, and conversely, MISP was detectable in the samples immunoprecipitated with the OIP5 antibody (Figure 7B–D), suggesting the formation of a MISP-OIP5 complex.

**Figure 8:**
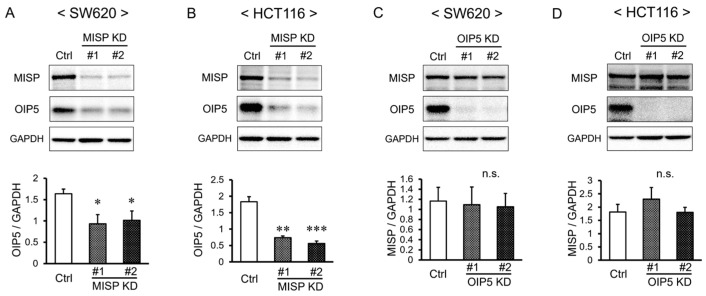
Interrelationship between the expression levels of MISP and OIP5. (**A**,**B**) Analysis of changes in OIP5 expression resulting from decreased MISP expression in (**A**) SW620 and (**B**) HCT116 cells. (**C**,**D**) Investigation of the impact of reduced OIP5 expression on MISP expression levels in (**C**) SW620 and (**D**) HCT116 cells. Each set of experiments was conducted with three replicates (*n* = 3). * *p* < 0.05; ** *p* < 0.01; *** *p* < 0.001; n.s., no significant difference.

Further analysis using Western blotting was conducted to investigate the impact of MISP on OIP5 expression levels. The reduction in MISP expression through knockdown (MISP KD) led to a significant decrease in OIP5 expression levels (Figure 8A,B). We also evaluated whether OIP5 influences MISP expression levels and found that reducing OIP5 expression through knockdown (OIP5 KD) did not affect the expression levels of MISP (Figure 8C,D).

### 2.8. Reduction in MISP Expression Decreases the Levels of Phosphorylated STAT3 in Colorectal Cancer Cells

**Figure 9:**
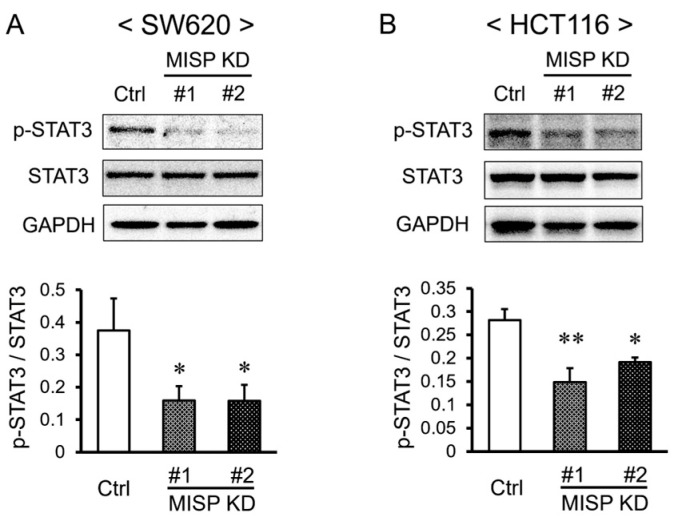
Effect of MISP on JAK2-STAT3 signaling in human CRC cells. (**A**,**B**) Investigation of how decreased MISP expression affects phosphorylated STAT3 levels in (**A**) SW620 and (**B**) HCT116 cells. Each experimental condition was replicated three times (*n* = 3). * *p* < 0.05; ** *p* < 0.01.

Prior studies have indicated that the suppressed expression of OIP5 results in decreased levels of phosphorylated STAT3 (Tyr705) in the JAK2-STAT3 signaling pathway [28]. Building on this finding, we examined the levels of phosphorylated STAT3 in MISP KD cells. The results revealed a significant reduction in the levels of phosphorylated STAT3 (Tyr705) relative to total STAT3 in the MISP KD samples compared to the control group (Figure 9A,B).

### 2.9. Rescue of Reduced Cell Proliferation through OIP5 Overexpression in the Context of Decreased MISP Expression

**Figure 10:**
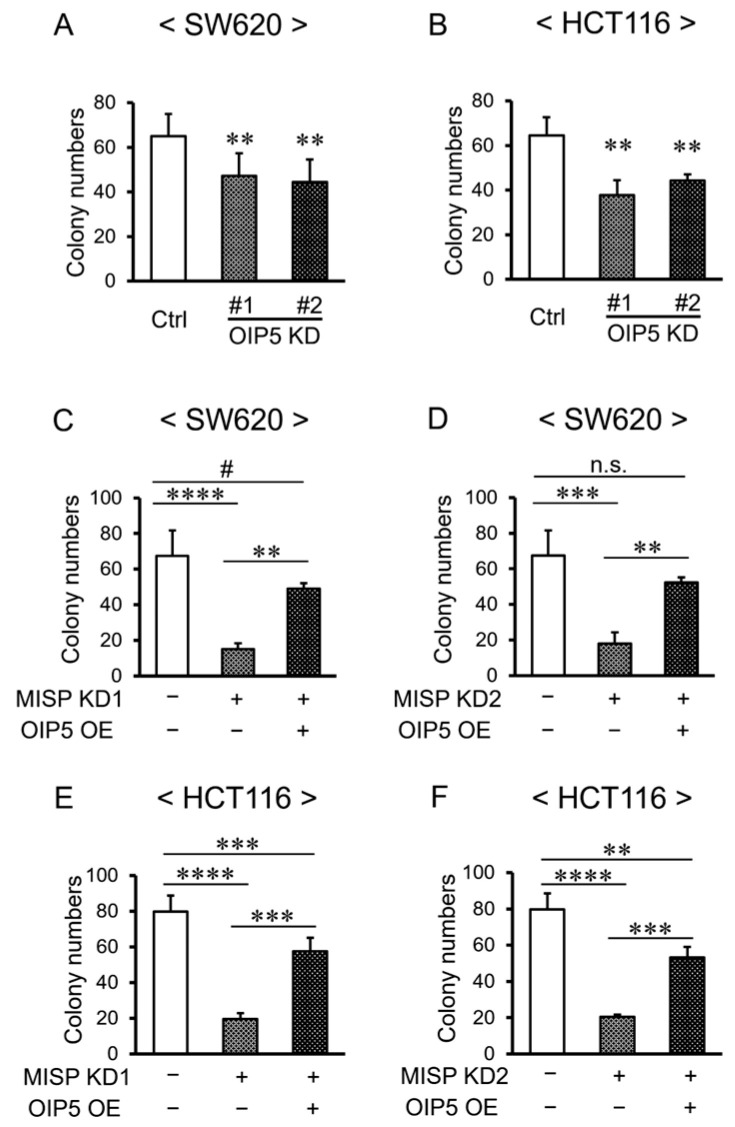
Effect of OIP5 on cell proliferation in human CRC cells. (**A**,**B**) Investigation of the effect of reduced OIP5 expression on cell proliferation in (**A**) SW620 and (**B**) HCT116 cells. (**C**–**F**) Assessment of the impact of OIP5 overexpression on cell proliferation under reduced MISP expression in (**C**,**D**) SW620 and (**E**,**F**) HCT116 cells. Each experiment was conducted with four replicates (*n* = 4). # *p* < 0.1 (indicating a trend); ** *p* < 0.01; *** *p* < 0.001; **** *p* < 0.0001; n.s., no significant difference.

Next, we aimed to determine whether the decreased cell proliferation observed in cases of reduced MISP expression could be attributable to a corresponding decrease in OIP5 expression. To assess the impact of OIP5 on cell proliferation, we conducted a colony formation assay. The knockdown of OIP5 (OIP5 KD) led to a decrease in colony formation in SW620 and HCT116 cell lines, indicating a reduced proliferative capacity (Figure 10A,B). Subsequently, we transfected cells in order to induce MISP knockdown (MISP KD) either alone or in combination with OIP5 overexpression (MISP KD + OIP5 OE) in SW620 and HCT116 cells. The colony formation assay revealed a significant enhancement in cell proliferation in the MISP KD + OIP5 OE group compared to the MISP KD group. However, in SW620 cells, there was either no significant difference or a trend towards decreased proliferation, while in HCT116 cells, a notable decrease in cell proliferation was observed in the MISP KD + OIP5 OE group when compared to the control (Figure 10C–F).

## 3. Discussion

MISP is found on the apical membrane of the colon, and its absence has been shown to aggravate DSS-induced colitis. While colitis is a known risk factor for CRC, the connection between MISP and CRC has not yet been explored. In this study, we aimed to elucidate the role of MISP in CRC development. Initially, we confirmed MISP expression levels in colorectal tissues from both healthy individuals and CRC patients (including primary tumors and adjacent normal tissues), utilizing the TCGA cancer genome database. Our findings revealed significantly elevated levels of MISP in both primary tumors and adjacent normal tissues of CRC patients compared to healthy controls. Interestingly, a bimodal distribution of MISP expression was observed in the colon tissues of healthy individuals, with the higher expression levels mirroring those observed in CRC patients’ tissues. However, no significant difference was found in MISP expression between primary tumors and adjacent normal tissues in CRC patients. In the subsequent analysis, we leveraged the GEPIA2 server (http://gepia2.cancer-pku.cn/, accessed on 19 January 2024) to evaluate the prognostic implications of MISP expression levels in CRC patients [29]. Despite the anticipatory hypothesis, our investigation revealed no statistically significant disparities in survival rates between patient cohorts characterized by high versus low MISP expression within the colon. Since it has been suggested that increased expression of MISP is caused by inflammation in the colon [18], these findings suggest that high expression of MISP in healthy subjects indicates a potential inflammatory state in the colon, and since its levels are comparable to those in patients with CRC, we considered MISP to be a risk factor for CRC.

Furthermore, we investigated the impact of reduced MISP expression on cell proliferation in human CRC cell lines using colony formation assays. The results showed that knocking down MISP led to a significant decrease in colony-forming capabilities, indicating that MISP facilitates CRC cell proliferation. It has been reported that increases in MISP are associated with decreased cell proliferation and expression of immune checkpoint molecules (resulting in tumor growth) and increased gene mutation burden [30]. Reducing MISP expression also inhibits cell proliferation in pancreatic ductal adenocarcinoma cells [30]. This aligns with the findings of our current study, which demonstrate that reducing MISP expression leads to decreased cell proliferation in CRC cells. These findings indicate that MISP promotes proliferation in human CRC cells and that elevated MISP expression in human colon tissue may be a risk factor for CRC development. Furthermore, we examined the effects of chronic inflammation in both WT and *Misp* KO mice using a colitis-induced colorectal tumor model. Our analysis revealed no significant differences in the rate of body weight change, DAI score, or survival rate between the two groups. It is important to note that intestinal diseases can be categorized as either acute, typically lasting a few weeks (usually between 7 to 14 days), or chronic, persisting for several months or longer [31]. These findings suggest that MISP may offer protection against acute inflammation in the colon [18] but does not appear to have the same effect on chronic inflammation. Subsequently, we assessed tumor development in the colons of *Misp* KO mice using a colitis-induced colorectal tumor model. This model, which involved AOM and multiple doses of DSS, is known to trigger chronic inflammation and subsequent tumor formation in the colon. These tumors begin as polypoidal growths and have a histopathology similar to that of human CRC, although they lack mucosal invasion and show few metastases [32]. Notably, there was a significant reduction in both the total number of colon tumors and tumors larger than 2 mm in *Misp* KO mice compared to WT mice. However, no significant difference was observed in the incidence of tumors smaller than 2 mm. These observations suggest that MISP may play a role in the initiation and progression of tumors in a colitis-induced colorectal tumor model.

Next, we focused on identifying factors related to MISP that contribute to the development of colorectal tumors. Initially, we compared gene expression changes in HCT116 cells between a control group and a group with MISP KD using RNA-seq analysis. This analysis revealed significant expression changes in 70 genes in the MISP KD group compared to the control. Furthermore, we utilized the HuRI database to identify proteins that potentially interact with MISP and genes that exhibit altered expression in our RNA-seq analysis. This search highlighted OIP5 as a protein associated with MISP. Notably, OIP5 has been previously reported to be highly expressed in various cancers, including CRC, hepatocellular carcinoma, renal cell carcinoma, bladder cancer, breast cancer, and glioma [33,34,35,36,37,38]. OIP5, also known as LINT-25, interacts with lamina-associated polypeptide 2α (LAP2α), a nuclear protein involved in chromatin binding during the cell cycle [39]. Like Mis18β, OIP5 is crucial for the structure and function of the centromere/kinetochore and accumulates specifically at centromeres during the telophase–G1 phase of the cell cycle [40]. The knockdown of OIP5 has been shown to trigger G1 arrest-dependent apoptosis and reduce cell proliferation in breast cancer cells [41]. Additionally, OIP5 KD has been observed to inhibit epithelial–mesenchymal transition (EMT) and downregulate phosphorylated JAK2 and phosphorylated STAT3 in the JAK2-STAT3 pathway in nasopharyngeal carcinoma [28]. STAT3, typically located in the cytoplasm, translocates to the nucleus upon phosphorylation of tyrosine 705 residues by cytokine signaling or tyrosine kinase-bearing oncoproteins, subsequently regulating the transcription of genes crucial for cell cycle progression and survival [41,42]. The knockdown of STAT3 using siRNA has been demonstrated to inhibit proliferation and invasion while inducing apoptosis in human CRC cells [43]. Thus, OIP5 appears to promote nasopharyngeal carcinoma progression by modulating the JAK2-STAT3 signaling pathway [28].

To investigate the relationship between OIP5 and CRC, we compared the expression levels of OIP5 in healthy individuals to those in CRC patients. We analyzed both primary tumors and adjacent normal tissues using data from TCGA, a comprehensive cancer genome database. Our findings showed that OIP5 expression was significantly higher in CRC patients compared to healthy subjects. Healthy subjects displayed a wide spectrum of OIP5 expression levels, ranging from low to high. These findings align with those from the MISP study. In CRC patients, primary tumors exhibited significantly higher OIP5 expression levels compared to the adjacent normal tissues. This suggests that the level of MISP expression might indicate the risk of tumorigenesis and that OIP5 might be specifically upregulated in tumorigenic cells. Furthermore, a correlation analysis of MISP and OIP5 expression in colorectal tissues from healthy individuals revealed a strong relationship between the expression levels of these two proteins. This indicates that MISP and OIP5 expression levels vary together in colorectal tissues. We next examined the cellular localization of MISP and OIP5 expression in vitro using immunofluorescence staining. In HCT116 cells, MISP was localized in the cytoplasm, whereas OIP5 was present in both the nucleus and cytoplasm. We also explored the interaction between MISP and OIP5 through immunoprecipitation experiments, using 293FT cells overexpressing MISP or OIP5. These experiments suggested the formation of a MISP/OIP5 complex. When MISP/OIP5 was overexpressed, endogenous OIP5 showed a strong presence in the input samples, indicating that the MISP/OIP5 complex might activate OIP5 mRNA expression. This was further supported by the detection of endogenous OIP5 through immunoprecipitation using an anti-OIP5 antibody, confirming high OIP5 expression in 293FT cells. Thus, the formation of the MISP/OIP5 complex appears to be a consistent event, regardless of OIP5 overexpression. Subsequently, we conducted Western blot analysis to evaluate changes in MISP and OIP5 protein expression levels in human CRC cells. We found that OIP5 expression significantly decreased following the knockdown of MISP in SW620 and HCT116 cells. In contrast, MISP expression levels did not change significantly upon knocking down OIP5 expression in the same cell lines. These results imply that OIP5 and MISP share a similar expression pattern in both healthy and CRC-affected colon tissues, with a high correlation in expression levels. Additionally, the co-localization of MISP with OIP5 in the cytoplasm of CRC cells suggests that MISP may unidirectionally regulate OIP5 expression by forming a complex with it in the cytoplasm. In other words, the increased expression of OIP5 may be caused by MISP, whose expression is increased by subclinical inflammation in healthy subjects and may be a cause of tumorigenesis.

**Figure 11:**
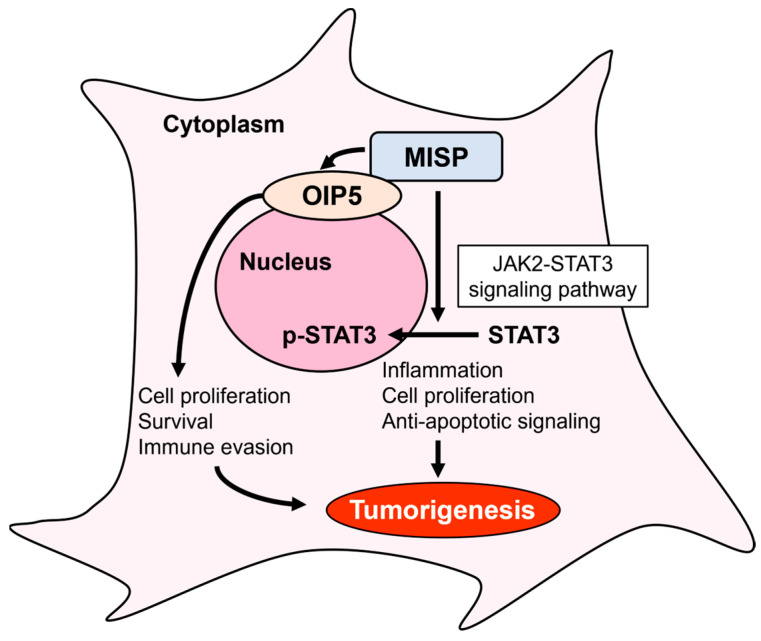
Schematic diagram of signaling pathways involving MISP. MISP promotes colorectal tumorigenesis by upregulating phosphorylated STAT3 in the JAK2-STAT3 signaling pathway, in addition to its interaction with OIP5.

To explore the mechanisms underlying intestinal tumorigenesis in relation to MISP, we examined the JAK2-STAT3 signaling pathway, which has been previously reported to be promoted by OIP5 [28]. Our observations revealed a significant reduction in the expression of STAT3, specifically in its phosphorylated form at the tyrosine 705 residue, following MISP knockdown in SW620 and HCT116 cells. These findings suggest that MISP facilitates the activation of the JAK2-STAT3 signaling pathway by increasing the levels of phosphorylated STAT3. As mentioned earlier, targeting the JAK2-STAT3 signaling pathway is a promising therapeutic strategy for CRC. This approach aims to intervene in crucial mechanisms associated with cancer progression and survival. Therefore, MISP has considerable potential as a therapeutic target for CRC, given its involvement in the JAK2-STAT3 pathway. Therefore, inhibiting MISP via nucleic acid therapeutics such as low-molecular-weight inhibitors, antisense nucleotides, and siRNA could be effective in CRC development and progression through two pathways: OIP5, which may regulate critical cellular processes involved in CRC progression such as cell proliferation, survival, and immune evasion, and the JAK2-STAT3 axis, which promotes cancer-promoting activities such as inflammation, proliferation, and anti-apoptotic signaling (Figure 11). However, verification of MISP-related signaling in the mouse colon was not possible in this study. Consistency between in vitro and in vivo findings remains a challenge for future research.

In order to determine if the reduced cell proliferation accompanying decreased MISP expression was related to OIP5 expression levels, we conducted a cotransfection experiment involving MISP KD and OIP5 overexpression (OE). The results indicated that the decrease in cell proliferation observed with MISP knockdown in SW620 and HCT116 cells was significantly counteracted by OIP5 OE. However, in the HCT116 cell line, cell proliferation was notably reduced in the group transfected with both MISP KD and OIP5 OE, compared to the control group without MISP KD or OIP5 OE. This suggests that the decrease in proliferation caused by MISP knockdown was not completely reversed by OIP5 OE. The difference in cell proliferative capacity between HCT116 and SW620 used in this study may also account for the difference in changes in cell proliferative capacity caused by MISP KD. Therefore, it is likely that the reduction in proliferation induced by MISP deficiency is not solely mediated by a decrease in OIP5 expression but is influenced by multiple factors.

It is conceivable that another contributing factor could be a decrease in the expression of TGF-β1. Previously, we have found that *Misp* KO mice exhibit decreased TGF-β1 expression in their colonic mucosa [18]. TGF-β1 is recognized for its dual role, acting both as a tumor suppressor in moderately to highly differentiated primary tumors and as a tumor growth factor in metastatic CRC cells [44]. It forms complexes with SMAD and indirectly activates NF-κB, which then upregulates WNT5A expression [45]. WNT5A facilitates EMT by increasing Vimentin expression and reducing E-cadherin levels, both of which are markers associated with EMT [46]. Furthermore, colitis-induced intestinal tumors, triggered by AOM and DSS administration, accelerate progression by promoting EMT [47]. EMT is critically involved in the advancement and metastasis of CRC [48,49]. Therefore, the occurrence of CRC due to MISP may be partially attributed to the decreased expression of TGF-β1. In the future, further exploration of the roles of MISP and TGF-β1, along with EMT, in CRC could lead to a deeper understanding of the mechanisms underlying MISP-driven colorectal tumorigenesis.

## 4. Materials and Methods

### 4.1. Gene Expression Analysis Utilizing the Cancer Genome Database

Through the UCSC Xena Browser (https://xenabrowser.net, accessed on 8 February 2021) [20,21,22], we conducted a comparative analysis of MISP and OIP5 gene expression levels in colorectal tissues. This comparison was between healthy individuals and cancer patients, encompassing both primary tumor and adjacent normal tissues. The data for this comparison were obtained from TCGA, a comprehensive cancer genome database. Specifically, the TCGA TARGET GTEx dataset, available at https://ocg.cancer.gov/programs/target (accessed on 8 February 2021), was used to compare the expression of MISP in healthy subjects and CRC patients.

### 4.2. Cell Culture

The human CRC cell lines SW620 and HCT116 were obtained from the American Type Culture Collection (Manassas, VA, USA). These cell lines were cultured in Dulbecco’s Modified Eagle Medium (DMEM; Wako, Tokyo, Japan). The medium was supplemented with 10% fetal bovine serum (FBS) (HyClone, Logan, UT, USA), 100 units/mL penicillin, and 100 μg/mL streptomycin. The cells were incubated at a constant temperature of 37 °C in a humidified chamber with 5% CO_2_.

### 4.3. Transfection of siRNA into Human CRC Cell Lines

The knockdown of MISP and OIP5 was achieved by transfecting siRNA into SW620 and HCT116 cell lines. The cells were plated at a density of 5.6 × 10^5^ cells per well in 12-well plates. The siRNAs (80 pmol/well; Thermo Fisher Scientific, Waltham, MA, USA) included those targeting MISP (HSS133913 and HSS133914) and OIP5 (HSS117619 and HSS174278). A negative control was prepared using Mission siRNA (80 pmol/well) (SIC-001; Sigma-Aldrich, St. Louis, MO, USA). The siRNA transfection was conducted using Lipofectamine^®^ 3000 (Thermo Fisher Scientific), and the experiments were performed 48 h after the transfections. The groups were labeled as follows according to the siRNA transfected: HSS133913, MISP KD1; HSS133914, MISP KD2; HSS117619, OIP5 KD1; and HSS174278, OIP5 KD2.

### 4.4. Colony Formation Assay

To evaluate cell proliferation, SW620 and HCT116 cells transfected with specific siRNAs or plasmids were seeded at a density of 1.0 × 10^3^ cells per well in 12-well plates. After a period of 5 days, colonies exceeding a predetermined size threshold (SW620, 100 µm; HCT116, 200 µm) were counted.

### 4.5. Ethical Statement

All animal experiments were conducted in strict accordance with the ARRIVE guidelines and the Japanese Act on Welfare and Management of Animals. The research adhered to Kitasato University’s Regulations for the Care and Use of Laboratory Animals. The protocol for animal experimentation was approved by the President of Kitasato University and by the Institutional Animal Care and Use Committee of Kitasato University (Approval ID: 20-039). A humane endpoint was implemented for mice displaying signs of moribundity or experiencing severe weight loss (a loss of 20% of body weight within a few days).

### 4.6. Animals

*Misp* KO mice, which were developed in the C57BL/6J strain, were generated as previously described [18]. The animal facilities were climate-controlled, maintaining an ambient temperature of 22 ± 2 °C and a relative humidity of 40–60%. The mice were housed under a 12 h light/12 h dark cycle and had access to standard laboratory feed CE-2 (CLEA Japan, Tokyo, Japan) and tap water ad libitum.

### 4.7. Colitis-Induced Colon Tumor Mouse Model

In this study, 8-week-old male WT or *Misp* KO mice were used. Each mouse received an intraperitoneal injection of 10 mg/kg azoxymethane (AOM) (A5486; Sigma-Aldrich). Five days after the AOM administration, 2.5% (*w*/*v*) DSS (molecular weight 36,000–50,000 Da; MP Biomedicals, Santa Ana, CA, USA) was provided in the drinking water for 5 days, followed by 14 days of regular tap water. This cycle of DSS administration was repeated three times in total. Seven days after the last DSS cycle, the mice were euthanized, and their colons were collected. Throughout the experiment, body weight measurements and observations of stool characteristics were conducted every 2–3 days. Weight changes and DAI scoring were performed as previously described [18]. Mice that met the humane endpoint criteria before the end of the study were euthanized, and survival curves were generated to compare survival rates. The collected colons were fixed in 10% formalin overnight at 4 °C. Tumors in the colons were counted using a stereomicroscope and categorized based on their size: total, smaller than 2 mm, and larger than 2 mm.

### 4.8. Exploration of MISP-Related Factors in Colorectal Tumorigenesis

To investigate factors associated with MISP in colorectal tumorigenesis, we performed RNA-seq. RNA was extracted from control and MISP-KD1 HCT116 cells using NucleoSpin^®^ RNA (Takara Bio, Shiga, Japan). The extracted RNA was then subjected to RNA-seq analysis by GENEWIZ on the Illumina NovaSeq platform (2 × 150 bp configuration, 6 Gb per sample; Azenta, Chelmsford, MA, USA). Enrichr and KEGG Mapper were used to perform pathway analysis on genes whose expression was significantly changed by MISP KD in order to identify pathways associated with MISP. In addition, we utilized the Human Reference Interactome (HuRI) database, which identifies protein–protein interactions (PPIs) through the yeast two-hybrid (Y2H) method [27], to explore potential MISP-related proteins among the candidate genes identified in the RNA-seq analysis.

### 4.9. Gene Expression Correlation Analysis

We conducted a correlation analysis on the expression levels of the MISP and OIP5 genes using the UCSC Xena Browser (https://xenabrowser.net, accessed on 1 March 2022). This analysis utilized RNA-seq data obtained from healthy colon tissue samples available in TCGA. Specifically, we utilized the TCGA TARGET GTEx dataset, which can be accessed at https://ocg.cancer.gov/programs/target (accessed on 1 March 2022).

### 4.10. Immunofluorescence Staining

HCT116 cells were seeded in µ-Slide 8-well chambers (ibidi, Gräfelfing, Germany) at a density of 1.5 × 10^4^ cells per well. Immunofluorescence staining was performed 24 h after seeding. The cells were fixed with 4% paraformaldehyde for 10 min at room temperature and permeabilized by treating with 0.1% Triton X-100 in PBS for 10 min at room temperature. Non-specific binding was blocked by incubating the cells with 5% goat serum for 30 min at room temperature. The cells were then incubated with either rabbit polyclonal anti-MISP antibody (1:100 dilution; 26338-1-AP, Proteintech, Rosemont, IL, USA) or rabbit polyclonal anti-OIP5 antibody (1:50 dilution; 12142-1-AP, Proteintech) for 1 h at room temperature. Subsequently, the cells were incubated with Alexa Fluor 488-conjugated goat anti-rabbit IgG secondary antibody (1:500 dilution; A11034, Cell Signaling Technology, Danvers, MA, USA) for 1 h at room temperature. Finally, the cells were mounted in ProLong Diamond Antifade Mountant with DAPI (Thermo Fisher Scientific) and imaged using a Floid Cell Imaging Station (Thermo Fisher Scientific).

### 4.11. Western Blot Analysis

Protein levels in SW620 and HCT116 cells, transfected with either the negative control, MISP KD, or OIP5 KD construct, were assessed using Western blot analysis. The method employed was exactly as previously described [18]. Primary antibodies used included rabbit polyclonal anti-MISP antibody (1:1000 dilution; 26338-1-AP, Proteintech), rabbit polyclonal anti-OIP5 antibody (1:1000 dilution; 12142-1-AP, Proteintech), rabbit monoclonal anti-STAT3 antibody (D3Z2G) (1:1000 dilution; #12640, Cell Signaling Technology), rabbit monoclonal anti-phospho-Stat3 antibody (Tyr705) (D3A7) (1:1000 dilution; #9145, Cell Signaling Technology), and HRP-conjugated mouse monoclonal anti-GAPDH antibody (1:5000 dilution; HRP-60004, Proteintech). The secondary antibody used was peroxidase-conjugated anti-rabbit IgG antibody (1:1000 dilution; #7074, Cell Signaling Technology).

### 4.12. Construction of the MISP Expression Plasmid

RNA was extracted from HCT116 cells using TRI reagent (Molecular Research Center, Cincinnati, OH, USA). This was followed by reverse transcription to synthesize cDNA using the ReverTra Ace kit (TOYOBO, Osaka, Japan). To clone the MISP gene product, TA cloning was performed using the TA PCR Cloning Kit (Biodynamics, Tokyo, Japan). The resulting cloned MISP gene product (REFSEQ: accession NM_173481.4) was then inserted downstream of the CAG promoter of the pCAGGS vector [50].

### 4.13. Investigation of the Interaction between MISP and OIP5 through Immunoprecipitation

To study the interaction between MISP and OIP5, we transfected 293FT cells with MISP expression plasmid and OIP5 expression plasmid (RG202255, ORIGENE, Rockville, MD, USA) using Lipofectamine^®^ 3000 (Thermo Fisher Scientific). A non-transfected group served as the control. Four groups were established: control, MISP overexpression, OIP5 overexpression, and co-overexpression of MISP and OIP5. Cells were treated with 200 µL of RIPA lysis buffer (ATTO, Tokyo, Japan), 2 µL of protease inhibitor, and 2 µL of phosphatase inhibitor (both from ATTO), followed by incubation at 4 °C for 15 min and centrifugation at 15,000 rpm for 10 min at 4 °C to extract proteins. The proteins were then immunoprecipitated using the Dynabeads Protein A Immunoprecipitation Kit (Veritas, Tokyo, Japan) following the manufacturer’s protocol. Rabbit polyclonal anti-MISP antibody (3 µL, 26338-1-AP, Proteintech) and rabbit polyclonal anti-OIP5 antibody (5 µL, 12142-1-AP, Proteintech) were used for immunoprecipitation. The samples were denatured and eluted with 2% SDS and 5% 2-mercaptoethanol, subjected to SDS-PAGE, and transferred onto a hydrophobic polyvinylidene fluoride membrane (PALL, Port Washington, NY, USA). To prevent non-specific binding, the membrane was incubated with Bullet Blocking (Nacalai Tesque, Kyoto, Japan) for 5 min at room temperature. Overnight incubation at 4 °C was performed with rabbit polyclonal anti-MISP antibody (1:1000; 26338-1-AP, Proteintech) or rabbit polyclonal anti-OIP5 antibody (1:1000; 12142-1-AP, Proteintech). This was followed by a 1 h incubation at room temperature with peroxidase-conjugated anti-rabbit IgG antibody (1:1000; #7074, Cell Signaling Technology). Subsequently, the luminescence reaction was carried out using ECL prime Western Blotting Detection Reagents (Cytiva, Marlborough, MA, USA) and detected with the Omega Lum C Imaging System (Gel Company, San Francisco, CA, USA).

### 4.14. Statistical Analysis

The results are presented as mean values ± standard deviation. Student’s *t*-test was employed to determine significant differences between the two groups. When comparing multiple groups, the Tukey–Kramer test was utilized. Pearson’s product-moment correlation coefficient was used for correlation analyses. A *p*-value of less than 0.1 was considered to indicate a trend, while a *p*-value below 0.05 was considered to indicate a statistically significant difference. All statistical analyses were conducted using the EZR statistical software (version 1.62) [51].

## 5. Conclusions

In conclusion, MISP enhances colorectal tumorigenesis through its interaction with OIP5 and stimulates colorectal tumor growth by upregulating the levels of phosphorylated STAT3 in the JAK2-STAT3 signaling pathway. These findings suggest that suppressing MISP expression levels has an inhibitory effect on CRC development and may be a valuable approach to understanding the mechanisms of colitis-induced colorectal tumorigenesis.

## Data Availability

The detailed data of the current study are available from the corresponding authors upon reasonable request.
